# Characterization of complete chloroplast genome of endemic species of Korea Peninsular, *Salvia chanryoenica* (Lamiaceae)

**DOI:** 10.1080/23802359.2018.1495115

**Published:** 2018-10-17

**Authors:** Young-Ho Ha, Kyoung Su Choi, Kyung Choi

**Affiliations:** Division of Forest Biodiversity, Korea National Arboretum of the Korea Forest Service, Pochen, 1186, Korea

**Keywords:** *Salvia chanryoenica*, Lamiaceae, chloroplast genome, phylogenetic analysis, endemic species

## Abstract

*Salvia chanryoenica* is an endemic species, which locates on the ridges of mountains in South Korea. In this study, we determined the complete chloroplast (cp) genome sequence of *S. chanryoenica*; cp genome of *S. chanryoenica* is 151,689 bp in length and consists of a large (82,903 bp) and small (17,634 bp) single-copy regions, separated by a pair of identical inverted repeats (25,576 bp). This genome contains unique 79 protein-coding genes, 30 tRNA, and 4 rRNA. The gene order and organization of the *S. chanryoenica* are consistent with those of other Lamiaceae cp genomes. The overall GC content of the whole genome was 37.9%. Phylogenetic tree constructed based on 71 protein-coding genes demonstrated a sister relationship within genus *Salvia*.

The genus *Salvia* L. is a member of Lamiaceae, which comprises approximately 900 species with a worldwide distribution. It also has economic and medicinal value known as oriental medicine in Central Asia (Zhou et al. 2005; Zhong et al. 2009). Three species of *Salvia* (*S. plebeia, S. japonica*, and *S. chanroenica*) and classification of the genus have been reported from Korea. Based on the life cycle, morphological characters such as the number of leaves, shapes, and hair of plants (Donoghue 2002) are determined. *Salvia chanryoenica* is an endemic species, which locates on the ridges of mountains in South Korea (Chung et al. 2017). Although this plant has economic utility and importance as a genetic resource, molecular studies have not been conducted. Strategic research and conservation of important genetic resources are important. Chloroplast (cp) genomes are significantly resourced such as structural diversity (Sinn et al. 2018), molecular markers (Wang et al. 2018), and biogeographical inference (Ha et al. 2018). In this study, we reported the complete cp genome sequence of *S. chanryoenica* using next-generation sequencing technology. The plastid genome will contribute to develop protection strategy for this Endangered species.

*S. chanryoenica* was collected from Mt. Sobaek, South Korea. The voucher specimen was deposited at the herbarium of Korea National Arboretum (KH). Total genomic DNA was extracted using a DNeasy Plant MiniKit (Qiagen Inc., Valencia, CA, USA) and used for the next-sequencing with Illumina Miseq plat (Illumina, San Diego, California, USA). For the genome annotation, the online software DOGMA (Wyman et al. 2004) and tRNAscan-SE (Schattner et al. 2005) were used to adjust its difference by comparison with related genomes.

The cp genome of *S. chanryoenica* (Genbank accession number MH261357) is 151,689 bp in length and consists of a large (82,903 bp) and small (17,634 bp) single-copy regions, separated by a pair of identical inverted repeats (25,576 bp). This genome contains 79 unique protein-coding genes, 30 tRNA, and 4 rRNA. 18 genes encoded introns among unique genes of *S. chanryoenica*, among which 12 are protein-coding genes and six are tRNA genes. Three protein-coding genes include two introns (*clpP*, *ycf3,* and *rps12*), and the overall C + G content of *S. chanryoenica* is 37.9%. The gene order and organization of the *S. chanryoenica* are consistent with those of other Lamiaceae cp genomes.

Phylogenetic analysis was conducted using a gene data matrix consisting of 71 protein-coding genes from 15 in Lamiaceae (Figure 1). *Erythranthe lutea* and *Paulownia tomentosa* were used as an outgroup. Maximum likelihood tree was constructed with RAxML (Stamatakis 2014) using the GTR + R+I model with 1000 bootstrap replicates. The genus *Salvia* was well-supported monophyletic (100% bootstrap values, BS) and *S. chanryoenica* was sister to *S. miltiorrhiza*. Our results, the cp genome sequence of *S. chanryoenica* may contribute to a better understanding of the evolution of *Salvia.* Also offers a useful resource for molecular marker and species conservation.

**Figure 1.**
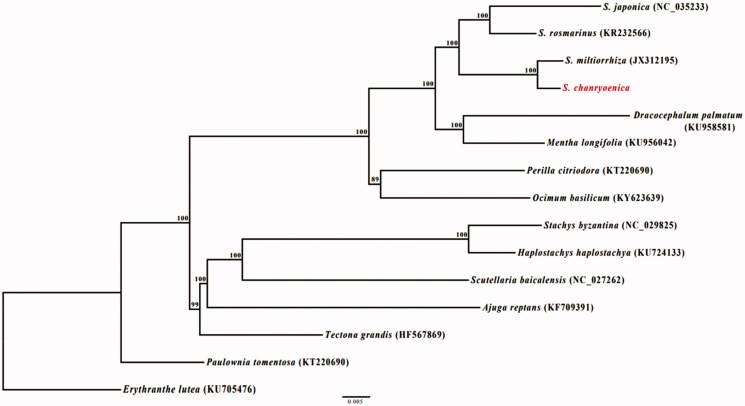
Maximum-likelihood phylogenetic tree of *S. chanryoenica* with 14 species belonging to the Lamiaceae based on chloroplast protein-coding sequences. Numbers in the nodes are the bootstrap values from 1000 replicates.
